# A combinatorial deep learning method for Alzheimer’s disease classification-based merging pretrained networks

**DOI:** 10.3389/fncom.2024.1444019

**Published:** 2024-10-17

**Authors:** Houmem Slimi, Ala Balti, Sabeur Abid, Mounir Sayadi

**Affiliations:** Research Laboratory SIME, ENSIT, University of Tunis, Tunis, Tunisia

**Keywords:** Alzheimer’s disease (AD), pretrained networks (PN), evaluation metrics (EM), SMOTE, data augmentation (DA)

## Abstract

**Introduction:**

Alzheimer’s disease (AD) is a progressive neurodegenerative disorder characterized by cognitive decline, memory loss, and impaired daily functioning. Despite significant research, AD remains incurable, highlighting the critical need for early diagnosis and intervention to improve patient outcomes. Timely detection plays a crucial role in managing the disease more effectively. Pretrained convolutional neural networks (CNNs) trained on large-scale datasets, such as ImageNet, have been employed for AD classification, providing a head start for developing more accurate models.

**Methods:**

This paper proposes a novel hybrid deep learning approach that combines the strengths of two specific pretrained architectures. The proposed model enhances the representation of AD-related patterns by leveraging the feature extraction capabilities of both networks. We validated this model using a large dataset of MRI images from AD patients. Performance was evaluated in terms of classification accuracy and robustness against noise, and the results were compared to several commonly used models in AD detection.

**Results:**

The proposed hybrid model demonstrated significant performance improvements over individual models, achieving an accuracy classification rate of 99.85%. Comparative analysis with other models further revealed the superiority of the new architecture, particularly in terms of classification rate and resistance to noise interference.

**Discussion:**

The high accuracy and robustness of the proposed hybrid model suggest its potential utility in early AD detection. By improving feature representation through the combination of two pretrained networks, this model could provide clinicians with a more reliable tool for early diagnosis and monitoring of AD progression. This approach holds promise for aiding in timely diagnoses and treatment decisions, contributing to better management of Alzheimer’s disease.

## 1 Introduction

Alzheimer’s disease (AD) is a degenerative neurological condition marked by cognitive decline, behavioral abnormalities, and memory loss. In order to manage the illness and enhance the patients’ quality of life, early diagnosis is essential. However, because the structural alterations in Alzheimer’s disease are subtle and complex, precisely identifying the condition using MRI scans remains a substantial issue. Brain MRI pictures show subtle structural differences that are difficult to interpret without sophisticated tools, and conventional diagnostic procedures frequently fail to capture these complex patterns. In order to support healthcare practitioners, more sophisticated, dependable, and automated classification systems are required. This is because early and accurate diagnosis might be challenging.

The complex patterns in MRI scans that differentiate between different stages of Alzheimer’s disease are difficult for traditional machine learning models and even some deep learning architectures to grasp. Issues including overfitting, computational efficiency, and cross-dataset generalization are not sufficiently addressed by many of the models that are currently in use. More advanced models are therefore required in order to efficiently learn and generalize these patterns, resulting in classifications that are more accurate. Though promising in other medical imaging tasks, advanced deep learning models are still limited in their ability to classify AD patients because of the particular difficulties presented by the disease’s course and its subtle impacts on brain structure. This justifies the exploration of novel model architectures and combinations to push the boundaries of what is currently achievable in AD diagnosis.

In this paper, we present a combinatorial deep learning method that combines DenseNet121 and Xception, two potent pretrained networks. DenseNet121’s dense connection makes feature reuse and gradient flow more effective. Because every layer in DenseNet121 is feed-forward coupled to every other layer, the vanishing gradient issue is lessened and feature reuse is encouraged, which enhances learning and performance. Conversely, Xception lowers model parameters and improves computational performance thanks to its depthwise separable convolutions. The convolution procedure is broken down into two parts in this architecture: a depthwise convolution and a pointwise convolution. This results in a significant reduction in computational cost without sacrificing performance. By combining these two networks, we aim to leverage the strengths of both architectures, resulting in a model that is both powerful and efficient.

Our strategy produced excellent results, with an average improvement in accuracy of 10% over the previous approaches. The effective fusion of Xception’s efficient convolutions and DenseNet121’s dense connectivity is responsible for this improvement. By using the Synthetic Minority Over-sampling Technique (SMOTE), which addressed the dataset’s class imbalance problem, this performance improvement was further improved. In order to balance the dataset without just copying existing samples, SMOTE creates synthetic samples for the minority class by interpolating between existing samples. The model can learn more efficiently thanks to this balanced dataset, particularly when some stages of Alzheimer’s disease are underrepresented in the training set.

Considerable progress has been made in the categorization of Alzheimer’s disease from MRI scans using the suggested combinatorial strategy. Utilizing the advantages of both Xception and DenseNet121, our model offers a reliable and effective solution. While Xception’s computational efficiency enables faster and more resource-efficient training and inference, DenseNet121’s dense connections enable a better and more thorough grasp of the subtle aspects of MRI images. SMOTE integration makes the model more robust in real-world applications by improving its generalization across imbalanced datasets. These developments open the door to earlier and more precise Alzheimer’s disease diagnosis, which may improve patient outcomes and facilitate the development of more potent disease management techniques. This study highlights the potential of combinatorial deep learning approaches in overcoming existing limitations and sets the stage for future research in this critical area.

This paper is structured as follows: we explain in section 2 some State-Of-the-Art Models, the dataset, preprocessing steps, followed by overviews. Some mathematical formulations of the proposed model are described in Section 3 as well as the proposed model architecture. In Same section, the proposed model’s architecture will be presented. Section 4 details the results achieved in this study. In Section 5, we discuss the results. Finally, the implications of our findings and future directions for research are exposed. Our approach demonstrates the potential of the proposed hybrid model in enhancing Alzheimer’s research, providing a framework that can be extended to other medical imaging applications.

## 2 Related work

In the domain of medical image analysis, transfer learning (TL) has received a lot of attention. One example of this is its use in the image classification of the Alzheimer’s Disease dataset. In a related study, You et al. (2020) Developed a cascade neural network that utilizes both gait and EEG data for AD classification, significantly outperforming other methods with a three-way AD classification accuracy of 91.07%. The next year, Mohammed et al. (2021) proposed many machine learning algorithms and deep learning models to classify images of OASIS and Alzheimer’s disease Datasets, they achieved an average accuracy equal to 94%. Furthermore, a recent study by Al Shehri (2022) proposed a deep learning-based solution using DenseNet-169 and ResNet-50 CNN architectures for the diagnosis and classification of Alzheimer’s disease. The DenseNet-169 architecture achieved training and testing accuracy values of 0.977 and 0.8382, respectively, while ResNet-50 had accuracy values of 0.8870 and 0.8192. One study by Thayumanasamy and Ramamurthy (2022) evaluated various machine learning and deep convolutional architectures for detecting Alzheimer’s disease (AD) from mild cognitive impairment (MCI), Accuracy equal to 82.2% was achieved by using DenseNet169. In a recent study by Bamber and Vishvakarma (2023) presents a model using a shallow convolution layer in a convolutional neural network for Alzheimer’s disease diagnosis in image patches, boasting a high accuracy rate of 98%. Another study by Mahmud et al. (2023) evaluate approach on a dataset of MRI scans from patients with AD and healthy controls, achieving an accuracy of 95% for combined ensemble models. Raza et al. (2023) focus on segmenting and classifying Magnetic Resonance Imaging (MRI) scans of Alzheimer’s disease, their approach involves leveraging transfer learning and customizing a convolutional neural network (CNN), accuracy achieved 97.84%. In same year, Balaji et al. (2023) create a hybrid deep learning model based on CNN and LSTM architectures, to classify images of two datasets, they also performed segmentation to improve results, accuracy of 98.5% is achieved. A recent study by Ching et al. (2024) focus on using EfficientNet-B0 to classify AD images and reached accuracy equal to 87.17%. In same year, Ali et al. (2024) uses Fuzzy C-means technique for image segmentation and merge LSTM architecture with CNN one to classify brain images, they reached an accuracy of 98.13%. In this study, we propose to integrate Xception and DenseNet121, two newly modified pretrained deep learning networks. At 99.85%, the suggested model has the highest accuracy. These works show how transfer learning can be used to improve the accuracy of image classification in the Alzheimer’s dataset and show how transfer learning can be used to further develop deep learning-based techniques.

## 3 Database study and preprocessing

**FIGURE 1:**
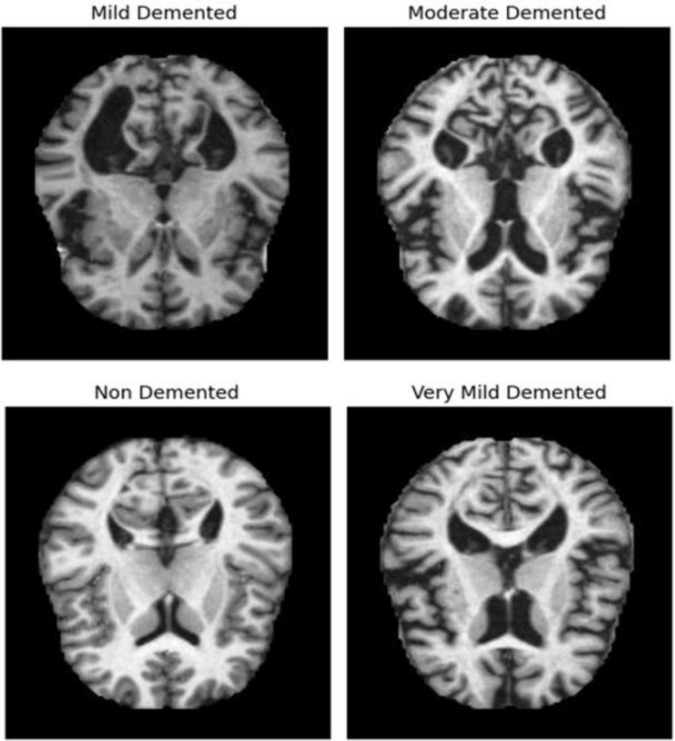
Example of images belongs to four classes from Alzheimer’s dataset.

The dataset of MRI images (link in Data Availability statement) initially consists of two parts: Training and Testing images, each with over 5000 images classified according to the severity of Alzheimer’s disease. Figure 1 shows some AD images categorized into four classes: Very Mild Demented, Mild Demented, Moderate Demented, and Non-Demented. Using the same methodologies as the proposed architecture, a comparison study with other pretrained networks has been conducted to assess the performance of the suggested model. All the dataset images were preprocessed as follows:

– Splitting data: the dataset contains two files: train and test. In this study, we merge the two parts and split obtained data into ratios of 80% for training and 20% for testing.
– Resizing Images: All images were resized to reduce computational power consumption and speed up application execution. By standardizing the image dimensions, we ensured that the model could process the data more efficiently, leading to faster training times without compromising accuracy.
– Data Augmentation: Data augmentation techniques were employed to create new training datasets that are variations of the original images. This process helps prevent overfitting by exposing the model to a wider variety of data. The augmentation methods used include:
  • Rotation: Rotating images helps the model become invariant to the orientation of the MRI scans, allowing it to learn features regardless of image alignment.
  • Flipping (Horizontal and Vertical): Flipping images increases data diversity by creating mirror images, which helps the model learn to recognize features from different perspectives.
  • Shifting (Width and Height): Shifting images horizontally and vertically helps the model become invariant to small positional changes in the MRI scans.
  • Zoom: Applying zoom augmentation ensures the model can handle variations in the size of brain structures, helping it focus on different levels of detail.
  • Brightness Adjustment: Adjusting brightness variations makes the model robust to changes in lighting conditions, ensuring consistent performance across different MRI scans.

These augmentation techniques not only increase the quantity of training data but also enhance the model’s ability to generalize by learning from a more diverse set of images. This diversity helps improve the model’s robustness and accuracy in identifying features relevant to different stages of Alzheimer’s Disease.

– Oversampling with SMOTE: The Synthetic Minority Oversampling Technique (SMOTE) was used by Chawla et al. (2002) and applied in this study to address the issue of unbalanced classes. SMOTE generates synthetic samples for the minority class by interpolating between existing samples.

### 3.1 Transfer learning technique

A powerful deep learning method called transfer learning (TL) uses model parameters that have previously been trained on a large dataset (such as the 1000-class ImageNet Dataset). Pretrained weights are also used in this strategy to speed up learning and improve accuracy in our model. It’s basically the same as imparting knowledge that one person has acquired to another. Instead than concentrating only on training the Fully Connected layers while maintaining the CNN layer unaltered, all layers are learned throughout the transfer learning process. Training just the CNN layers (either fully or partially) is one way to improve the model’s performance. This technique is used in this study by choosing two pretrained networks which are: DenseNet121 (Gupta and Mesram, 2022) and Xception (Chollet, 2017). The aim of this study is to merge these two PN, as will be described later, to improve the classification process.

### 3.2 Mathematical Formulations

• Performance metrics
• TP: True Positives
• TN: True Negatives
• FP: False Positives
• FN: False Negatives
• For the hybrid model:
• *Acc*_*Hybrid*_: Accuracy of the hybrid model

(1) $${\text{Acc}}_{\text{Hybrid}}=\frac{{\text{TP}}_{\text{Hybrid}}+{\text{TN}}_{\text{Hybrid}}}{{\text{TP}}_{\text{Hybrid}}+{\text{TN}}_{\text{Hybrid}}+{\text{FP}}_{\text{Hybrid}}+{\text{FN}}_{\text{Hybrid}}}$$

If *Acc*_*Hybrid*_ is higher than *Acc*_*DenseNet121*_ and *Acc*_*Xception*_, it indicates that the hybrid model is more accurate in its predictions. The hybrid model combines features from both DenseNet121 and Xception, potentially leveraging their complementary strengths, which can result in improved classification performance compared to each individual model. Thus, a higher accuracy for the hybrid model shows that it has a better overall performance in correctly identifying samples, proving its superiority over the individual models.

• Overall Performance Improvement
• For the hybrid model:
• ΔAcc_Hybrid_ : Improvement in accuracy of the hybrid model

(2) $$\Delta \,{\text{Acc}}_{\text{Hybrid}}={\text{Acc}}_{\text{Hybrid}}-\max\left({\text{Acc}}_{\mathrm{DenseNet121}},\mathrm{Ac}\,{\text{c}}_{\text{Xception}}\right)$$

The improvement in Δ*Acc*_*Hybrid*_ measures how much the accuracy of the hybrid model exceeds that of the best-performing individual model (either DenseNet121 or Xception). By subtracting the maximum accuracy of the individual models from the accuracy of the hybrid model, we can quantify the enhancement in performance provided by the hybrid approach. A positive value of indicates that the hybrid model offers superior accuracy compared to the single best model, demonstrating its effectiveness in improving classification results beyond what each individual model alone can achieve.

• Concatenation of Output Layers
• O: Output layer
• For the hybrid model:
• *O*_*Hybrid*_: Output layer of the hybrid model
• *O*_*DenseNet121*_: Output layer of DenseNet121
• *O*_*Xception*_: Output layer of Xception
• ⊕: Concatenation operation

(3) $${\text{O}}_{\text{Hybrid}}={\text{O}}_{\mathrm{DenseNet121}}⊕{\text{O}}_{\text{Xception}}$$

• Ensemble Performance Gain
• For the hybrid model:
• *G*_*Acc*_ : Gain in accuracy

(4) $$G_{A\,c\,c}=\frac{A\,c\,c_{H\,y\,b\,r\,i\,d}-A\,c\,c_{D\,e\,n\,s\,e\,N\,e\,t\,121}+A\,c\,c_{H\,y\,b\,r\,i\,d}-A\,c\,c_{X\,c\,e\,p\,t\,i\,o\,n}}{2}$$

The ensemble performance gain *G*_*Acc*_ calculates the average improvement in accuracy provided by the hybrid model compared to each individual model. It is determined by averaging the differences in accuracy between the hybrid model and DenseNet121, and between the hybrid model and Xception. This calculation helps quantify the extent to which the hybrid model outperforms both individual models. A positive *G*_*Acc*_ value signifies that the hybrid model achieves better accuracy than either DenseNet121 or Xception, thereby demonstrating the advantages of combining the strengths of both models. This gain highlights the effectiveness of the hybrid approach in improving classification accuracy beyond what is achieved by each individual model alone.

• Error Reduction
• E: Error rate
• *Acc*: Accuracy
• For the hybrid model:
• Δ*E*: Error reduction

(5) $$\Delta \,\text{E}=\min\left({\text{E}}_{\mathrm{DenseNet121}},{\text{E}}_{\text{Xception}}\right)-{\text{E}}_{\text{Hybrid}}$$

(6) E=1-A⁢c⁢c⁢u⁢r⁢a⁢c⁢y

Error reduction Δ*E* measures how much the hybrid model’s error rate is reduced compared to the best-performing individual model. It is calculated by subtracting the error rate of the hybrid model from the minimum error rate of DenseNet121 and Xception. This metric helps to quantify the improvement in classification performance of the hybrid model by showing that it has a lower error rate than the best individual model. A lower error rate in the hybrid model indicates enhanced accuracy and effectiveness in classification, providing evidence that combining the two models leads to better overall performance compared to relying on either model alone. This reduction in error underscores the advantage of the hybrid approach in minimizing misclassifications.

• Statistical Significance
• For the hybrid model:
• *D*_*Acc,i*_ : Difference in accuracy for the i-th sample
• $\bar{D_{A\,c\,c,i}}$ : Mean difference in accuracy
• *s*_*D,Acc*_ : Standard deviation of the differences in accuracy
• *t*_*Acc*_ : t-value for accuracy

(7) $${\text{D}}_{\mathrm{Acc},i}={\text{Acc}}_{\mathrm{Hybrid},i}-\max\left({\text{Acc}}_{\mathrm{DenseNet121},i},\mathrm{Ac}\,{\text{c}}_{\mathrm{Xception},i}\right)$$

(8) $$\bar{{\text{D}}_{\text{Acc}}}=\frac{1}{\text{n}}\,\sum_{\text{i}=1}^{\text{n}}{\text{D}}_{\mathrm{Acc},i}$$

(9) $${\text{s}}_{D,\mathrm{Acc}}=\sqrt{\frac{1}{n-1}\,\sum_{\text{i}=1}^{\text{n}}{\left({\text{D}}_{\mathrm{Acc},i}-\bar{{\text{D}}_{\text{Acc}}}\right)}^{2}}$$

(10) $${\text{t}}_{\text{Acc}}=\frac{\bar{{\text{D}}_{\text{Acc}}}}{{\text{s}}_{D,\mathrm{Acc}}/\sqrt{\text{n}}}$$

To determine the statistical significance of the hybrid model’s performance improvement, we calculate several key metrics. First, *D*_*Acc,i*_ measures the accuracy difference between the hybrid model and the best individual model for each sample, showing how much better the hybrid model performs on a sample-by-sample basis. The mean difference in accuracy $\bar{D_{A\,c\,c}}$ averages these differences across all samples, indicating overall improvement. The standard deviation of these differences *s*_*D,Acc*_ assesses the variability of the improvements, with a low value suggesting consistent superiority. Finally, the t-value *t*_*Acc*_ assesses the statistical significance of the mean accuracy improvement by comparing $\bar{D_{A\,c\,c}}$ to its standard error. A high t-value confirms that the observed improvement in accuracy is statistically significant, demonstrating the hybrid model’s robustness and superiority over the individual models.

### 3.3 The model structure

**FIGURE 2:**
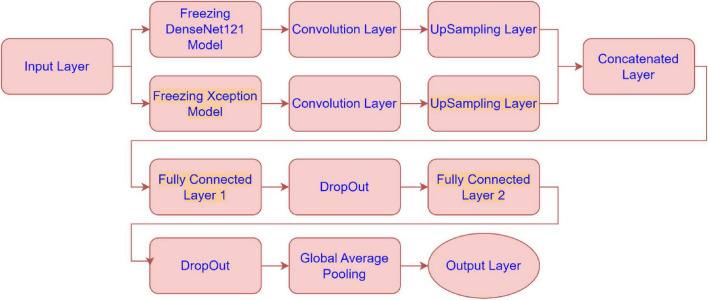
Architecture of the proposed model.

The architecture combines feature extraction from these pre-trained models with additional convolutional layers, upsampling, and fully connected layers to optimize the model’s performance. Below is a detailed explanation of each sub-module in the architecture presented by Figure 2:

• Freezing DenseNet121 Model:

The DenseNet121 model is used as a feature extractor, but its weights are “frozen”, meaning they are not updated during training. This allows the model to leverage pre-trained weights without modifying them. The output of the frozen DenseNet121 model is passed to the next convolutional layer.

• Freezing Xception Model:

Similarly, the Xception model is also frozen and used as a feature extractor. The frozen model’s output is sent to a convolutional layer.

• Convolution Layer (for DenseNet121 and Xception):

After extracting features from both DenseNet121 and Xception, these features are further processed by convolutional layers. The convolution layers apply filters to extract spatial hierarchies from the feature maps. These layers help in refining the features obtained from the pre-trained models.

• UpSampling Layer (for DenseNet121 and Xception):

Once features are processed through the convolution layers, the UpSampling layers increase the resolution of the feature maps (essentially scaling them up). This is likely done to match the spatial dimensions of the two models’ feature maps before concatenating them.

• Concatenated Layer:

The outputs from the upsampled DenseNet121 and Xception models are concatenated along the channel dimension. This step fuses the feature representations from both models, combining their strengths to form a more comprehensive feature set for the next stage of the model.

• Fully Connected Layer 1:

After concatenation, the fused feature map is passed through a fully connected (dense) layer. This layer serves to learn and capture more abstract relationships in the combined features, transforming the spatial features into more compact, higher-level representations.

• DropOut:

Dropout is applied to prevent overfitting by randomly setting a fraction of the input units to 0 during training. This forces the model to not rely on any one specific feature and improves generalization.

• Fully Connected Layer 2:

Another fully connected layer follows the dropout. This layer further transforms the features, likely preparing them for the final classification. It’s part of the final layers that learn high-level representations to differentiate between the classes.

• Global Average Pooling:

Global average pooling reduces the spatial dimensions of the feature maps by averaging them across each channel. This reduces the dimensionality of the output, producing a single value per feature map (or channel). It replaces traditional fully connected layers before the output, promoting model generalization and reducing the risk of overfitting.

• Output Layer:

The final output layer produces the class probabilities for the input data. This layer takes the reduced representation from the global average pooling layer and outputs a prediction, likely using softmax activation for multi-class classification in Alzheimer’s disease MRI image classification.

## 4 Results

Several evaluation criteria, including accuracy, precision, recall, F1-score, and AUC, are used to assess how well deep learning architecture’s function.

Where the percentage of accurate predictions to all occurrences analyzed is measured by the Accuracy metric. The positive patterns that are accurately predicted from all of the projected patterns in a positive class are measured using the precision metric. The fraction of positive patterns that are correctly categorized is measured by recall, and the harmonic mean of recall and accuracy values is represented by the F1score metric. These evaluation metrics are more explained by Hossin and Sulaiman (2015).


(11) $$R\,e\,c\,a\,l\,l=\frac{T\,P}{T\,P+F\,N}$$

(12) $$P\,r\,e\,c\,i\,s\,i\,o\,n=\frac{T\,P}{T\,P+F\,P}$$

(13) $$F\,1\,S\,c\,o\,r\,e=2.\frac{P\,r\,e\,c\,i\,s\,i\,o\,n.R\,e\,c\,a\,l\,l}{P\,r\,e\,c\,i\,s\,i\,o\,n+R\,e\,c\,a\,l\,l}$$

(14) $$A\,c\,c\,u\,r\,a\,c\,y=\frac{T\,N+T\,P}{T\,N+T\,P+F\,N+F\,P}$$

The Area Under the Curve (AUC) is an effective metric having values in the interval [0, 1]. Since there is perfect discrimination between instances of the two classes, the AUC is equal to 1. Conversely, when all Benign cases are classified as Malignant, the AUC equals 0 and vice versa.

**FIGURE 3:**
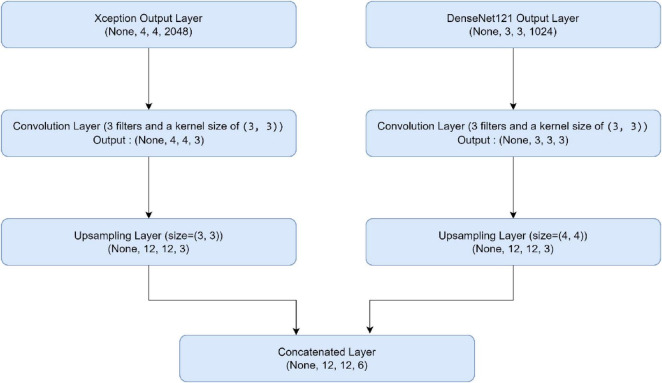
Feature fusion architecture.

**FIGURE 4:**
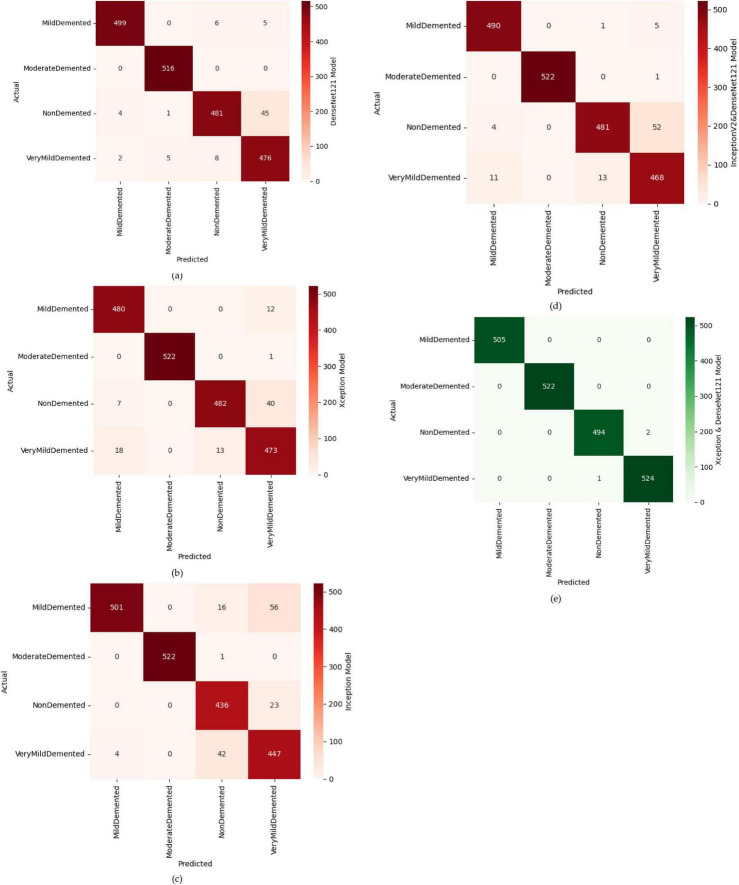
Confusion matrices of different models, where **(a)** confusion matrix of DenseNet121 model, **(b)** confusion matrix of Xception model, **(c)** confusion matrix of inception model, **(d)** confusion matrix of InceptionV2&DenseNet121 model, **(e)** confusion matrix of Xception&DenseNet121 model.

In this part, we use Kaggle platform with GPU P100 service for training and testing our proposed model to increase the running time of our code. The computer used for the experiments includes the following features: Windows 10 Professional, 64-bit operating system, Intel(R) Core(TM) i7-8750H CPU @ 2.20 GHz 2.21 GHz 8 GB Memory, x64-based processor. The specific approach is started by resizing images to 120x120 format. The next step consists of applying Data Augmentation on Alzheimer’s Dataset to get accurate results, this stage is followed by using the SMOTE Technique to solve the problem of imbalanced classes (The “Moderate Demented” class contains only 52 images). After that, we apply freezing on the first 40 layers of DenseNet121 and Xception models (freeze technique is used to make architecture more robust and to avoid overfitting), then merging them as explained in “The novel planned strategy” Part. The “Adam” optimization (Kingma and Jimmy, 2014) applied and learning rate parameter is fixed to 0.0001, the loss function is “categorical_crossentropy”, the activation function chosen is “RELU’ in hidden layers and “Softmax” is applied to the output layer. The first Fully Connected layer have 2048 nodes and the second one have 1048 nodes, each one is followed by dropout layers (with dropout rate equal to 0.3). We use EarlyStopping (with “patience” parameter equal to 7), and L2 Regularization (with waited decay equal to 0.001) to avoid overfitting problem. Note that the number of epochs for train was fixed to 20. The performance of the suggested model in comparison to the normal and hybrid pretrained models is displayed in Table 1. Figure 1 illustrated the different classes into the dataset, while Figure 2 show the proposed model’s architecture with details also explained in sub section 3.3. According to Figure 3, the Xception model outputs a tensor of shape (None, 4, 4, 2048), while DenseNet121 produces a tensor of shape (None, 3, 3, 1024). To harmonize these feature maps, each output is passed through a convolutional layer with 3 filters of size (3, 3), reducing the channel dimensions to 3, resulting in tensors of shape (None, 4, 4, 3) for Xception and (None, 3, 3, 3) for DenseNet121. These are then upsampled to (None, 12, 12, 3) using factors of (3, 3) and (4, 4) respectively, aligning their spatial dimensions. Finally, the upsampled feature maps are concatenated along the channel axis, resulting in a fused tensor of shape (None, 12, 12, 6). Figure 4 show respectively the confusion matrixes of DenseNet121, Xception, InceptionV3, InceptionV3&DenseNet121 and Xception&DenseNet121 of Alzheimer’s Dataset. Take note that there are fewer False Positive and False Negative cases in the confusion matrix of Xception&DenseNet121.

**TABLE 1:** Evaluation metrics of different models.

	Accuracy (%)	F1Score (%)	AUC (%)	Precision (%)	Recall (%)	*P*-values
**Alzheimer’s dataset**						
InceptionV3	93	93.3	95.7	93.5	93.5	0.059
Xception	95.5	95.5	97	95.6	95.6	0.6
DenseNet121	96.2	96.2	97.5	96.3	96.3	0.19
DenseNet121&InceptionV3	95.7	95.7	97.2	95.8	95.8	0.45
**DenseNet121&Xception**	**99.85**	**99.85**	**99.9**	**99.85**	**99.88**	15 × 10^−12^

The bold values are the values of the proposed model.

**FIGURE 5:**
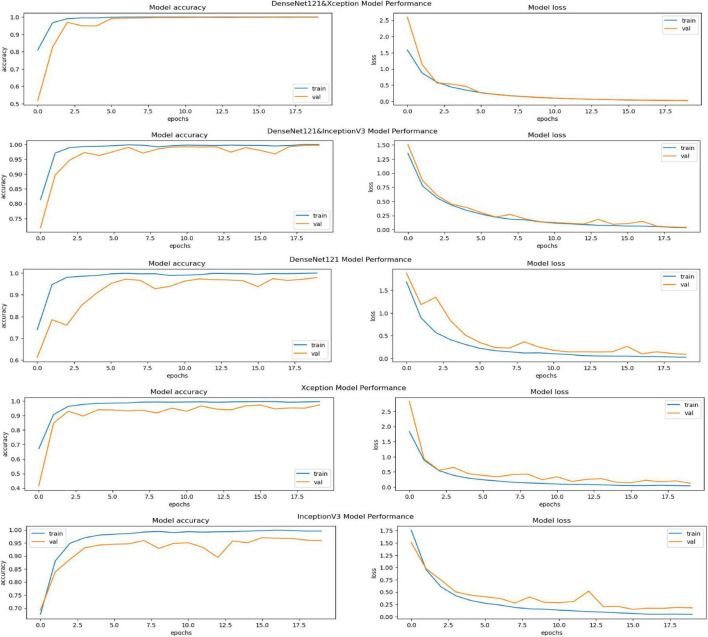
Accuracy and loss curves for different models.

**FIGURE 6:**
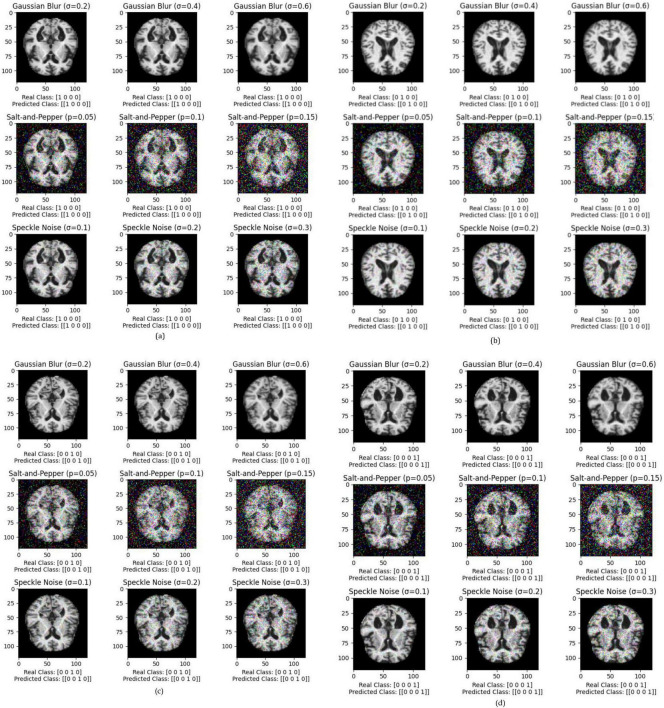
Exploring the impact of Gaussian Blur (σ = 0.2, 0.4, 0.6), salt-and-pepper noise (*p* = 0.05, 0.1, 0.15), and speckle noise (σ = 0.1, 0.2, 0.3) on Alzheimer’s dataset image classification for each class, where **(a)** impact of noises in Class 1, **(b)** impact of noises in Class 2, **(c)** impact of noises in Class 3, **(d)** impact of noises in Class 4.

**FIGURE 7:**
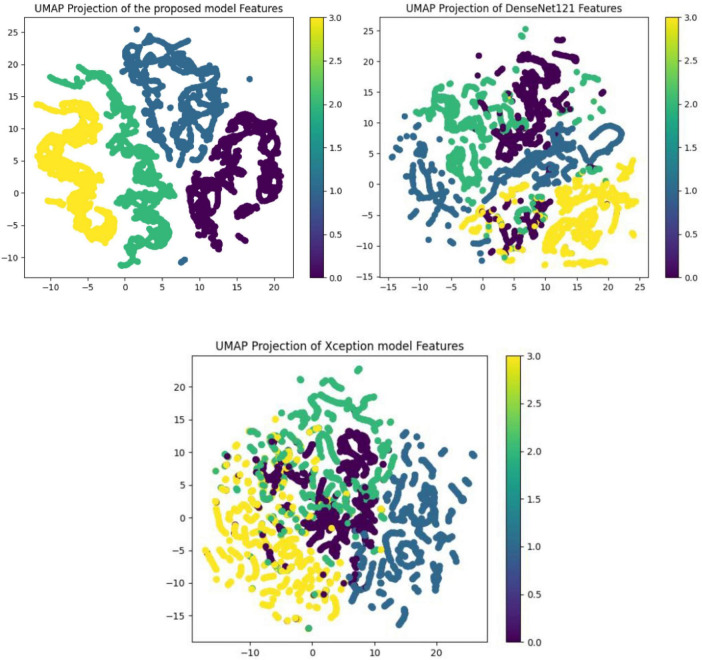
UMAP projection of extracted features: visualization of class separation in each model.

The performance of various models is greatly impacted by the critical usage of data augmentation strategies in this study. Figure 5 illustrated different curves of respectively accuracy and loss of each model in case of train and validation cases. Table 1 shows a comparison, in term of evaluation metrics, between different models: DenseNet121, Xception, InceptionV3, DenseNet121&InceptionV3 and DenseNet121&Xception. In Table 2, We offer an additional comparison in the AD Dataset between EM with and without the SMOTE approach. We compare results of the proposed model with some state-of-the-art ones in Table 3. In Table 4, we also make a comparison between our proposed model and some other models using the same dataset (MRI images from ADNI dataset). In Figure 6 and Table 5, we investigate the robustness of our Alzheimer’s dataset image classification model through the injection of various types of noise. The goal is to understand how different types and levels of noise affect the performance of the model across different classes. We applied three types of noise to the original images: Gaussian Blur with standard deviations (σ) of 0.2, 0.4, and 0.6 to simulate different levels of blurring, Salt-and-Pepper Noise with probabilities (p) of 0.05, 0.1, and 0.15 to mimic pixel corruption, and Speckle Noise with standard deviations (σ) of 0.1, 0.2, and 0.3 to simulate multiplicative noise. By experimenting with multiple parameter values for each type of noise, we aimed to observe their effects on the classification performance of our model. Our findings highlight the importance of evaluating model robustness against various types and levels of noise. Understanding the impact of noise on classification accuracy is crucial for developing more robust and reliable deep learning models for Alzheimer’s disease diagnosis. To ensure the robustness and generalizability of the proposed model, a 5-fold cross-validation approach was employed in Table 6. The dataset was randomly split into five subsets, and the model was trained and evaluated five times, each time using a different subset as the test set while the remaining four subsets were used for training. This process was implemented with shuffling enabled to ensure randomness and a fixed seed for reproducibility. After each fold, the accuracy was recorded, and the final performance was assessed by calculating the mean and standard deviation of the accuracy across all folds. The cross-validation yielded a mean accuracy of 99.72% and a standard deviation of 0.0141. To gain deeper insights into the discriminative power of the features extracted by the proposed model, dimensionality reduction techniques such as UMAP was applied. Specifically, features were extracted from the penultimate layer of the suggested model and then projected into a 2-dimensional space using UMAP (Uniform Manifold Approximation and Projection). UMAP was configured with 5 neighbors and a minimum distance of 0.3 to balance local and global structure in the data, while preserving the cluster structure. Figure 7 shows the resulting 2D scatter plot in the proposed model, in DenseNet121 model and the Xception model.

**TABLE 2:** Comparison between two proposed approaches (with and without SMOTE Technique).

	Accuracy (%)	F1Score (%)	AUC (%)	Precision (%)	Recall (%)
**Alzheimer’s dataset**					
Proposed approach (without SMOTE)	88.6	89.1	90.3	90	89.4
Proposed approach (with SMOTE)	**99.85**	**99.85**	**99.9**	**99.85**	**99.88**

The bold values are the values of the proposed model.

**TABLE 3:** Comparison between the proposed model and state of the art models.

References	Model name/type	Dataset used	Accuracy (%)
Nancy Noella and Priyadarshini, 2023	Bagged Ensemble Learning Classifier	AD−PD	90.3
Lubis et al., 2022	Naïve Bayes + Invariant Moment	AD MRI	94
Hussain and Shiren, 2023	SVM + Watershed Segmentation	ADNI	96.25
Shobha and Karthikeyan, 2024	AlexNet	AD MRI	94.5
Zhang et al., 2024	Attention Mechanism + GAN	MRI−PET	89.9
**The proposed Model 2024**	**DenseNet121+** **Xception**	**AD MRI**	**99.85**

The bold values are the values of the proposed model.

**TABLE 4:** Comparison between the proposed model and state of the art models applied on MRI images from ADNI dataset.

References	Model name/type	Accuracy (%)
Hazarika et al., 2023	Hybrid LeNet + AlexNet	93.58
Khan et al., 2023	Segmentation + Modified VGG	97.89
Balasundaram et al., 2023	Hippocampus Segmentation + CNN	94
Aborokbah, 2024	UNet + EfficientNet-B0	98.12
Esam and Mohammed, 2024	Modified CNN	97
Shaffi et al., 2024	Ensemble Classifier	96.52
Neethu and Roopa Jayasingh, 2024	MCLSO + SpinalNet	92.6
**The proposed model** **2024**	**DenseNet121+Xception**	**99.85**

The bold values are the values of the proposed model.

**TABLE 5:** Impact of various noise types on Alzheimer’s dataset image classification: all predicted classes match real classes.

Corresponding figure	Noise type and parameter
Figure 6a (Class 1)	Gaussian Blur (σ = 0.2, σ = 0.4, σ = 0.6)
	Salt-and-Pepper (*p* = 0.05, *p* = 0.1, *p* = 0.15)
	Speckle Noise (σ = 0.1, σ = 0.2, σ = 0.3)
Figure 6b (Class 2)	Gaussian Blur (σ = 0.2, σ = 0.4, σ = 0.6)
	Salt-and-Pepper (*p* = 0.05, *p* = 0.1, p = 0.15)
	Speckle Noise (σ = 0.1, σ = 0.2, σ = 0.3)
Figure 6c (Class 3)	Gaussian Blur (σ = 0.2, σ = 0.4, σ = 0.6)
	Salt-and-Pepper (p = 0.05, *p* = 0.1, p = 0.15)
	Speckle Noise (σ = 0.1, σ = 0.2, σ = 0.3)
Figure 6d (Class 4)	Gaussian Blur (σ = 0.2, σ = 0.4, σ = 0.6)
	Salt-and-Pepper (*p* = 0.05, *p* = 0.1, *p* = 0.15)
	Speckle Noise (σ = 0.1, σ = 0.2, σ = 0.3)

**TABLE 6:** Cross-validation technique.

	Fold1	Fold2	Fold3	Fold4	Fold5	Mean	Standard deviation
**Alzheimer’s Dataset**							
Cross-validation accuracies	99.66	99.85	99.74	99.65	99.7	99.72	0.0141

## 5 Discussion

The proposed hybrid model integrates DenseNet121 and Xception architectures, leveraging their unique strengths to improve the classification of Alzheimer’s disease using MRI images. The approach centers on freezing the pre-trained layers of these models to retain their pre-learned features while preventing the model from becoming overly complex or prone to overfitting. This combination of architectures is particularly appropriate for medical imaging tasks due to their proven success in domains such as breast and brain image classification. By freezing the pre-trained layers, the model benefits from the robust feature extraction capabilities of DenseNet121 and Xception while still allowing the new layers to adapt specifically to Alzheimer’s MRI data.

An essential enhancement to this approach is the application of SMOTE (Synthetic Minority Over-sampling Technique), which addresses class imbalance by generating synthetic examples for underrepresented classes. This results in a more balanced training dataset, allowing the model to better generalize across all classes. The model’s performance improvement, especially in correctly classifying minority classes, demonstrates the effectiveness of SMOTE in reducing bias and improving overall accuracy. This method enhances the model’s ability to identify complex features in the minority classes, which is crucial for a more equitable classification performance. Moreover, the model’s ability to remain robust under noisy conditions, such as Gaussian blur and salt-and-pepper noise, showcases its reliability for real-world medical imaging applications. Noise is an inherent challenge in medical images due to factors like equipment limitations or patient movement, and a model that can maintain high accuracy under these conditions is vital for clinical deployment. The robustness observed across various noise types highlights the model’s resilience, underscoring its potential for reliable clinical use. The hybrid model’s architecture, which balances DenseNet121’s dense connections and Xception’s computational efficiency through depthwise separable convolutions, contributes to an effective extraction and processing of features from MRI images. This synergy between the two models results in a powerful classifier that captures complex patterns while maintaining reasonable computational costs. Nevertheless, the increased complexity and resource demands present challenges, particularly in terms of training time and memory requirements. Future research could explore optimization techniques like pruning and quantization to reduce model size and improve efficiency without sacrificing performance.

In conclusion, the proposed hybrid model effectively capitalizes on the strengths of both DenseNet121 and Xception while addressing key challenges like class imbalance and noise robustness. While the model exhibits improved performance and robustness, future work should focus on further optimization and exploration of complementary architectures to enhance computational efficiency and scalability for broader clinical application.

## 6 Conclusion and recommendations

### 6.1 Conclusion

In this long work, we used a large Alzheimer’s disease (AD) dataset to carefully assess the performance of five different models: DenseNet121, Xception, InceptionV3, DenseNet121&InceptionV3, and DenseNet121&Xception. Beyond a simple evaluation, we also suggested new, altered architectures for DenseNet121, InceptionV3, MobileNetV2, and InceptionV3. The most notable accomplishment is the exceptional accuracy of our model, which achieved an astounding 99.85% inside the AD dataset. This notable enhancement marks a major advancement in our newly suggested architecture’s ability to classify and detect things. By utilizing these cutting-edge neural networks, we support the continuous endeavors to improve patient care and early AD diagnosis. Our results highlight the potential utility of transfer learning techniques in medical imaging, underscoring the significance of ongoing innovation in the battle against Alzheimer’s.

### 6.2 Recommendations

– **Further Validation on External Datasets:** Although the model performs remarkably well on the current AD dataset, it is essential to validate its generalizability by testing it on external and more diverse datasets, particularly those from different populations or imaging sources.
– **Incorporate Explainability Methods:** For clinical adoption, incorporating explainability techniques (e.g., Grad-CAM, SHAP) would help provide insights into the decision-making process of the model, ensuring transparency and fostering trust among healthcare professionals.
– **Optimization for Real-Time Deployment:** Investigating lightweight versions of the hybrid model could make it more suitable for real-time deployment in clinical settings where computational resources may be limited. This could involve pruning, quantization, or the integration of more efficient architectures like MobileNetV2.
– **Broader Clinical Applications:** Given the success in Alzheimer’s classification, the hybrid architecture could be adapted and tested for other neurodegenerative diseases, such as Parkinson’s and Huntington’s disease, thereby broadening its impact on early diagnosis across a range of conditions.
– **Ongoing Research in Transfer Learning:** Continuous refinement of transfer learning approaches should be pursued to stay ahead in the rapidly evolving field of medical imaging. Exploration of novel pre-training strategies on larger, more diverse medical datasets could further boost performance in specialized tasks.

## Data Availability

The datasets presented in this study can be found in online repositories. The names of the repository/repositories and accession number(s) can be found below: https://www.kaggle.com/datasets/tourist55/alzheimers-dataset-4-class-of-images.
